# Mechanobiological Implications of Cancer Progression in Space

**DOI:** 10.3389/fcell.2021.740009

**Published:** 2021-12-08

**Authors:** Hyondeog Kim, Yun Shin, Dong-Hwee Kim

**Affiliations:** ^1^ KU-KIST Graduate School of Converging Science and Technology, Korea University, Seoul, South Korea; ^2^ Division of Life Sciences, College of Life Sciences and Biotechnology, Korea University, Seoul, South Korea; ^3^ Department of Integrative Energy Engineering, College of Engineering, Korea University, Seoul, South Korea

**Keywords:** space mechanobiology, cellular mechanoadaptation, mechanotransduction, microgravity, cancer Progression

## Abstract

The human body is normally adapted to maintain homeostasis in a terrestrial environment. The novel conditions of a space environment introduce challenges that changes the cellular response to its surroundings. Such an alteration causes physical changes in the extracellular microenvironment, inducing the secretion of cytokines such as interleukin-6 (IL-6) and tumor growth factor-β (TGF-β) from cancer cells to enhance cancer malignancy. Cancer is one of the most prominent cell types to be affected by mechanical cues via active interaction with the tumor microenvironment. However, the mechanism by which cancer cells mechanotransduce in the space environment, as well as the influence of this process on human health, have not been fully elucidated. Due to the growing interest in space biology, this article reviews cancer cell responses to the representative conditions altered in space: microgravity, decompression, and irradiation. Interestingly, cytokine and gene expression that assist in tumor survival, invasive phenotypic transformation, and cancer cell proliferation are upregulated when exposed to both simulated and actual space conditions. The necessity of further research on space mechanobiology such as simulating more complex *in vivo* experiments or finding other mechanical cues that may be encountered during spaceflight are emphasized.

## 1 Introduction

During interstellar transportation, astronauts are exposed to a variety of environmental challenges, such as irradiation, microgravity, and decompression. One study addressing mortality during the space mission for 301 astronauts indicated 53 deaths, of which cancer accounted for 30%, with secondary frequent cases following external causes (including aircraft, spacecraft, and automobile accidents) accounted for 38% (Reynolds et al., 2019). In particular, irradiation has been considered as a carcinogen. For example, one experiment utilizing a linear accelerator-generated X-ray revealed that it induced stiffening in breast cancer, with a significant increase in collagen production through activation of the Wnt signaling pathway (Yakavets et al., 2020). However, accelerator-based experiments were limited to single-ion beams at fixed energies, as most radiobiological studies are. According to the National Aeronautics and Space Administrate (NASA), there are three types of space irradiation: particles trapped in the Earth’s magnetic field, particles shot into space during solar flares (solar particle events), and galactic cosmic rays, all of which are ionizing irradiation. Among them, galactic cosmic rays (GCRs) or solar particle events (SPEs) are the main contributors to irradiation-induced pathogenesis, including cancer, and they consist of diverse ion species with a continuous range of energies, thus forming a radiation spectrum (Norbury et al., 2016). Unfortunately, facilities on Earth are unable to fully simulate the dynamic aspect of space radiation. Moreover, relatively little research has been conducted on carcinogenesis within a space environment using a systematic approach. Rather, the resultant cellular responses, such as proliferation, migration, and gene expression, generally have been measured without attention to the underlying mechanism.

While there are technical limitations, experimental techniques that can mimic the conditions of space have been recently developed. For instance, multiple artificial-gravity research system (MARS) has been applied to test mice behaviors (Shiba et al., 2017), where mouse cages mimicking the space environment, e.g., a transportation cage unit, habitat cage unit (HCU), centrifuge-equipped biological experiment capacity, and artificial-g-section capacity were newly developed to clarify the effect of partial gravity and microgravity on mouse activity. This research not only demonstrated that the additional gravity could prevent reduction in bone density and muscle mass but further provided a novel insight on the molecular pathways regulating the microgravity-dependent cellular processes. The rapidly growing application for dimensional (3D) *in vitro* culture system can be another promising experimental approach. Recently, 3D printing of compound cell scaffolding has shown the enhanced cellular experience of *in vitro* extracellular environmental conditions, which results in a sustained tissue regeneration (Garlet and Santos, 2014). Since the flight experiments have provided a broad range of relevant findings for the application of biomedical goals to muscle/bone neurology and physiology, a variety of innovative tools have been applied, which results in new perspectives on the accurate effect of microgravity on animal body. An experimental rodent system with the caging environment of flight hardware was studied to explore the health condition of extended spaceflight (Choi et al., 2020). By monitoring the targeted locomotion of the rodents through the entire habitat, this study provided novel insights on the onset of chronic stress during the extended space flight. Housing in the animal enclosure module in spaceflight could further provided this notion (Lloyd et al., 2013). Housing experimental technique of animal enclosure module space hardware mimics the space conditions, where rodents were housed in particularly designed cages termed animal enclosure module (AEM) equipped with the waste management and gravity-independent nutrient distribution systems as well as 12-h light/darkness cycle, Time-lapse monitoring of animal behaviors in this system enabled to test physiological alteration of animals such as trabecular mouse bone tissues.

In this review, the effects of environmental change, i.e., irradiation, microgravity, and decompression, from Earth to space on cancer are discussed in terms of mechanical stress. We focus on the microenvironment of cancer cells in various tissues and their physical properties that affect cell migration, proliferation, and invasion. Furthermore, we scrutinize the morphological behavior of cancer at a single-cell level, associating the mechanotransduction, in which certain molecules sense the microenvironment and mediate the response, to adaptation. Based on previous studies considered representative of the physiological response to these changes, we suggest a potential physiological mechanism that results in the generation of cancer during space missions.

## 2 Effect of Distinct Environmental Differences Between Earth and Space on Human Body

### 2.1 Reduced Gravitational Force

Human homeostasis is adapted to Earth’s gravity, which is 1 g (9.8 m/s^2^). For instance, the cardiovascular system (Hughson et al., 2018), nervous system (Kohn and Ritzmann, 2018), and bone and muscle mass (Miyamoto et al., 1998) tend to be upregulated as the gravitational force increases. Gravitational force in space is non-zero, but reduced to 10^–6^ fold as the orbiting spacecraft forms an accelerated environment, and therefore termed “microgravity” (Herranz et al., 2013). Exposure to decreased gravity reduces membrane viscosity, decreases the open-state probability of ion channels, and increases the threshold of peripheral nerve stimulation (Ritzmann et al., 2017).

**FIGURE 1:**
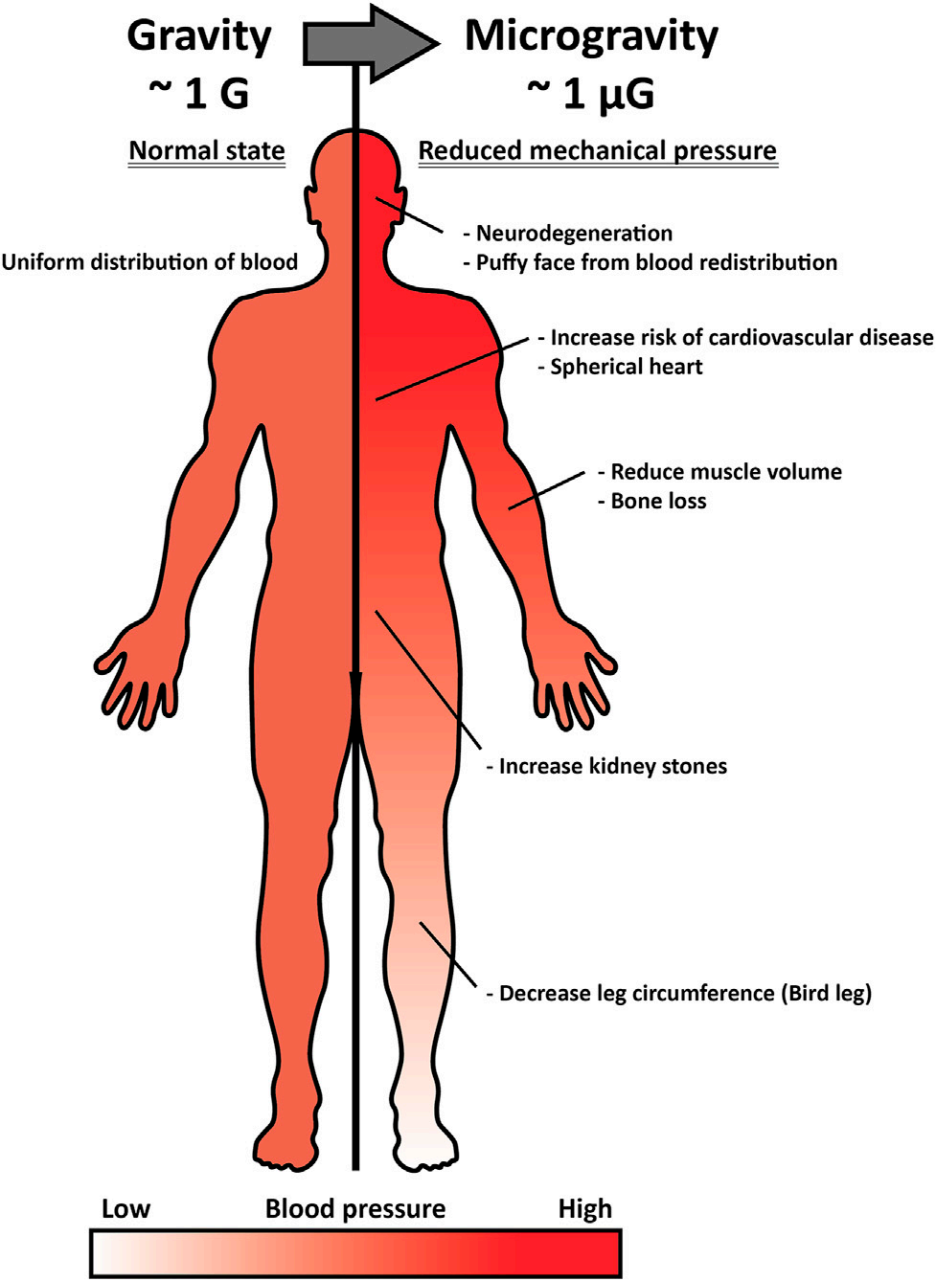
Inner pressure change by interstitial fluid shift under microgravity and its effect on the human body. Under normal gravitational force (∼1G), the pressure of interstitial fluid maintains a head-to-foot hydrostatic gradient (left). Under microgravity (∼1 µG), the interstitial fluid and pressure shifts upward, and such alteration induces optic disc stress, spherical heart, puffy face, kidney stone formation, bird leg, and muscle atrophy (right).

The concept of a head-to-foot hydrostatic gradient is not applied in space where gravity is considerably low. Without sufficient gravitational force, interstitial fluid is reduced by approximately 40% in the thigh and shifted to the head, altering the pressure of body parts (Baisch, 1993). Leg swelling generally occurs due to an abnormal interstitial fluid retention known as “edema,” inducing occlusion or compression and resulting in deep-vein thrombosis and inferior vena cava complication, among other complications. However, in space, where pressure on the head is higher and on the leg is lower than on Earth, forehead and facial tissue swelling occurs (Alexander et al., 2012). Such an interstitial fluid shift leads to spaceflight-associated neuro-ocular syndrome (SANS), which results in optic disc edema developed by prolonged exposure to microgravity (Figure 1) (Huang A. S. et al., 2019). Approximately 60% of astronauts experience impaired near and distant vision after long-term space missions, and five out of seven astronauts had disk edema and globe flattening after 6 months of long-term spaceflight, as assessed by a magnetic resonance imaging (MRI) scan (Mader et al., 2011). Space gravity-induced interstitial fluid shift could also increase the risk of kidney dysfunction and renal stone formation. Upward fluid shifts stimulate fluid-loss signal in the kidney and alter glomerular filtration by increasing the secretion of vasopressin, renin, and aldosterone, which have anti-diuresis, water reabsorption through the renin-angiotensin-aldosterone system, and sodium conservation functions, respectively (Liakopoulos et al., 2012). The serum of astronauts who participated in the Mir mission was extracted and analyzed, and the renin, vasopressin, and aldosterone concentrations were found to be consistently elevated during the entire metabolic ward period (Drummer et al., 2008). Combined with altered hormones and glomerular filtration, astronauts have a higher risk of proteinuria and calcium oxalate stone formation, as well as uric acid accumulation in post-flight, and a greater risk of calcium oxalate, calcium phosphate, and sodium urate stones during spaceflight.

Human muscles increase in volume and become stronger after exercise, e.g., lifting, loading 2–10 times the gravitational force (Harridge et al., 1998). However, several changes in muscle characteristics and function were altered after 6 months of long-term spaceflight (Lambertz et al., 2001). For example, maximal activation of the muscle decreased by 39%, and stiffness of the musculotendinous junction increased by 25%, resulting in an increase in impairment by limiting muscle movement (Lambertz et al., 2001). The decrease in muscle volume differed by body part, exhibiting −6% in calf and −3% in thigh, and decreased isometric and isokinetic strength in the knee, ankle, and elbow were observed in the range of −10.4 to −24.1%, −4 to −22.3%, and −7.5 to −16.7%, respectively (Gopalakrishnan et al., 2010). Recent evidence has shown that simulated or actual microgravity induces muscle atrophy in animals and humans by shifting myosin heavy chain (MHC) type I to II, thereby decreasing in muscle fiber size (Trappe et al., 2009). MHC is the major structural and contractile protein and consists of one slow phenotype (type I) and three fast phenotypes (type IIA, IIX, and IIB), depending on its twitching speed (Graziotti et al., 2001). The decrease in muscle mass due to transition of MHC type is termed slow-to-fast muscle fiber transition, and it has been observed in 5 and 11 days of short-term spaceflight (Edgerton et al., 1995). In 2001, it was suggested that muscle atrophy during spaceflight can be attributed to ubiquitination after the detection of 1.4- to 2.8-fold upregulated polyubiquitin mRNAs and ubiquitinated MHC (Ikemoto et al., 2001). When the rat soleus muscle was exposed to unweighted conditions, 55% of atrophy and 66% elevated protein breakdown were detected, with increased mRNA levels of the ubiquitin-conjugating enzyme E2, which plays a key role in the attachment of ubiquitin (Ub) to cellular proteins (Taillandier et al., 1996). Furthermore, 91 days of long-term spaceflight exhibited increased expression of muscle-specific E3 ubiquitin ligase, which recruits an E2 that has been loaded with ubiquitin, as well as the muscle RING-finger protein-1 (MuRF-1) and atrogin-1 (MAFbx), which are two genes of the ubiquitin-proteasome system (Sandona et al., 2012). Reduced muscle mass is directly associated with the risk of cardiovascular disease (CVD) (Srikanthan et al., 2016). Astronauts who participated in the Apollo lunar mission had 4–5 times higher CVD mortality risk than the non-flight control group, suggesting that exploration into deep space involves hazardous considerations (Delp et al., 2016).

Skeletal muscles excrete myokines such as myostatin and interleukins, which modulate the size of adipose tissue and inhibit proinflammatory adipokines that increases the risk of type 2 diabetes, cardiovascular disease, and cancer (Srikanthan et al., 2016; Sargolzaei et al., 2018). Muscle stimulation through physical exercises produces myokines that regulates autocrine, paracrine, and endocrine systems. Accordingly, muscular contractions extracts energy by spending glucose and lipids of white adipose tissue to balance whole-body metabolism, which, therefore negatively regulates the size of adipose tissues (Levy et al., 2018). However, myokine excretion is decreased as muscle-mass loss occurs under microgravity, and thus, the capacity of fat oxidation by skeletal muscles is reduced as well (Kelley, 2005). Since skeletal muscles take up fatty acids as fuel to provide the energy needed for movement and intestinal activity, reduction of muscle mass restrains the ability to inhibit triacylglycerol accumulation in adipocytes, leading to the incidence of obesity and related chronic diseases (Frayn, 2010; Barbat-Artigas et al., 2014).

### 2.2 Decompression

The atmospheric pressure within the troposphere decreases following the equation:

p(h)=p0e−ρ0ghp0=p0e−hH  H=p0ρ0g=RTMg=kTmg

where *p*(*h*), *p*_0_, *g*, *ρ*_0_, *R*, *T*, *M*, *m*, and *H* indicates the atmospheric pressure at altitude *h*, atmospheric pressure at *h* = 0, gravitational acceleration, mass density of air at 0 altitude, gas constant, temperature, average molar mass of dry air, molecular mass of ideal gas, and scale height, respectively (Berberan-Santos et al., 2010; Lente and Osz, 2020). This equation indicates that the overall atmospheric pressure decreases as the altitude increases, which also means that the partial pressure of oxygen is lower and causes hypobaric hypoxia (Du et al., 2019). For example, the atmospheric pressure and pO_2_ at average sea level, *h* = 0 m, is 101.3 and 21.3 kPa, respectively, while at the top of Mt. Everest, *h* = 8,850 m, is 33.7 and 7.1 kPa, respectively (West et al., 1983).

In outer space, where the altitude *h* is above 4 × 106 m, the atmospheric pressure falls below 10^–7^ kPa with the constituents of oxygen and nitrogen, as well as highly reactive oxygen and nitrogen (Horneck et al., 2010). Rapid elevation of altitude and decreased atmospheric pressure cause decompression sickness (DCS). DCS occurs when dissolved nitrogen (N_2_) in blood vessels evaporates, causing bubbles due to the decompressed atmosphere, which also occurs commonly to scuba divers. Evaporation expands the volume of N_2_ gas and damages or blocks the blood vessels, leading to neurological symptoms or pulmonary rupture in severe cases (Tawar and Gokulakrishnan, 2019). To prevent DCS, astronauts undergo decompression from 101 kPa to 70.3 Pa with a slightly increased percentage of oxygen at least 24 h before space exploration and breathe 100% of pure oxygen 1 hour before launch.

Hypobaric hypoxia, also known as high altitude hypoxia, is a condition in which deoxygenated blood is transferred to other organs, including the brain, due to a lack of both oxygen pressure and density, causing asphyxiation (Choudhury, 2018). Hypoxic conditions induce the production of reactive oxygen species (ROS) and increase oxidative stress. Reduced pO_2_ limits the availability of oxygen that functions as an electron acceptor inside the cell (Chen et al., 2018). The electron accumulation produces energy to excite the ground state of O_2_ and produces super oxide anion (·O_2_^−^), hydrogen peroxide (H_2_O_2_), and hydroxyl radicals (·OH^−^) (Chen et al., 2018). Another hypothesis connecting hypoxia with increased ROS is that low concentrations of nitric oxide (NO·) bind to cytochrome c oxidase and inhibit its function (Turrens, 2003). Therefore, the Michaelis constant, K_m_ for oxygen is increased and interferes with the electron transporter of terminal oxidase, resulting in ·O_2_^−^ formation at low oxygen concentration. Once deoxygenated tissues generates ROS, reperfusion of oxygen could induce the oxidative injury, generally termed ischemia-reperfusion injury (Chouchani et al., 2014). Hypoxia incudes ischemic succinate accumulation because oxidation into fumarate by succinate dehydrogenase is inhibited in the citric acid cycle. However, the accumulated ischemic succinate rapidly converts to fumarate by succinate dehydrogenase after reperfusion, which drastically generates ROS at mitochondrial complex I that is the main site of ROS production (Chouchani et al., 2014).

### 2.3 Radiation

Radiation is classified into two groups: non-ionizing radiation and ionizing radiation. Non-ionizing radiation is the low-frequency part of the electromagnetic spectrum that carries insufficient photon energy to cause ionization, which is lower than 10 eV, as defined by the International Commission on Non-Ionizing Radiation Protection (ICNIRP) (Ziegelberger and Icnirp, 2020). Non-ionizing radiation includes radiowave, microwave, near-infrared, and ultraviolet rays, which can adversely affect health, causing cataracts and corneal damage after expose for long periods (Wegener, 1994). The transmission power of radiation decreases as the wavelength becomes shorter and cannot permeate deeper. Therefore, ultraviolet (UV) rays cause diseases on the surface, such as keratitis and conjunctivitis, while visible and infrared rays cause cataracts, which have a deeper origin (Izadi et al., 2018). However, non-ionizing radiation is still considered safer than ionizing radiation (IR), which has a short wavelength and high frequency with energy higher than 13.8 eV, the energy required to ionize a substance. The quantity unit that indicates the absorbed dose of IR is Gray (Gy = Joule/kg) in the international system of units (SI) that measures the energy deposited by ionizing radiation in a unit mass of matter being irradiated (Mills, 2010). However, the degree of biological damage does not always correspond to the quantity of IR, but instead depends on factors such as linear energy transfer (LET) and type of radiation, such as gamma rays (X-rays), neutrons, or heavy ions, which determines the quality of IR (Hall and Giaccia, 2006). To measure the varying biological damage effect of IR, sievert (Sv) is the SI unit for absorbed dose equivalent, representing the equivalent dose and biologically effective dose of the deposit of a joule of radiation energy into a kilogram of human tissue (Hall and Giaccia, 2006).

The individual radiation exposure dose on Earth is approximately 3.0 millisieverts (mSv) annually because most radiation is screened out by Earth’s magnetic field and the atmosphere (Lerner and Gorog, 2021). However, during space exploration, astronauts are exposed to higher radiation by galactic cosmic rays (GCRs) and solar particle events (SPEs), resulting in a maximum effective dose of 150 mSv per half-year on the International Space Station (ISS) (Cucinotta, 2015). Since these radiations have low linear-energy transfer (LET), the amount of energy that an ionizing particle transfers to the material traversed per unit distance, their effect on human body are similar to gamma and X-rays, but containing higher charge and energy (HZE) particles and producing secondary neutrons that damage cells and tissues by inducing energy deposition (Cucinotta et al., 2001). GCR and SPE are high-energy protons and heavy ions, respectively, two types of IR. IR has the ability to remove electrons from their orbit directly and indirectly and cause oxidative damage to genetic materials, as well as oxidative metabolic stress (Azzam et al., 2012; Reisz et al., 2014). For instance, mitochondria, which consume up to 90% of oxygen in the body, are affected by ROS that damage the mitochondrial respiratory system, and such defects in mitochondrial functions result in rapid aging and pathological conditions, e.g., neurodegenerative and cardiovascular diseases and diabetes (Azzam et al., 2012). During long-term exposure to GCE and SPE, high-energy protons generate ROS by exciting cellular water, inducing circulatory disease and metabolic syndrome (Tapio et al., 2021). Furthermore, tissue fibrosis is a prominent side effect of IR exposure, which stimulates transdifferentiation into myofibroblasts that excessively secrete collagen types IV, V, and VI, as well as other extracellular matrix (ECM) proteins such as glycoproteins, fibronectin, laminin, and tenascin (Judge et al., 2015).

Edema is the second most common symptom of irradiation after fibrosis (Johansson et al., 2002). Radiation-induced tissue damage triggers acute inflammation and activates coagulation factor XII, the major protein that causes angioedema by increasing permeability and leakage of blood vessels (Dewald and Bork, 2006). Coagulation factor XII, also known as hemagen factor, in addition to its vascular coagulation functions, plays a central role in triggering the proinflammatory kallikrein–kinin system, leading to the formation of bradykinin, a peptide hormone that causes inflammation (Gobel et al., 2016). The thorax, salivary gland, brain, and neck are the major vulnerable points of radiation-induced swelling. This swelling leads to early injury of brain tissue and cognitive impairment within a day of irradiation, stimulating neuroinflammation, and it is characterized by somnolence, short-term memory loss, and attention deficits (Lumniczky et al., 2017). When tested in mice, acute exposure to ^16^O or ^48^Ti radiation particles significantly reduced cortical and hippocampal performance, causing a decrease in dendritic complexity and spine density (Parihar VK. et al., 2015). Radiation-induced degeneration exhibited comparable dose-dependent changes in the morphology of dendritic branches, number, and length of branch points (Parihar V. K. et al., 2015).

In addition to the effects of weightlessness, high-energy radiation in space can further contribute to bone loss. For example, IR stimulates early loss of bone vasculature by causing swelling, which directly disrupts blood flow to the bone and leads to avascular necrosis in severe cases (Watson and Adams, 2018). Furthermore, high-energy photon radiation has been connected to osteoporosis, causing an approximately 30% reduction in the mineral density of bone within 5 weeks of exposure to ionizing radiation of approximately 22.5 Gy or 45 Gy (Nishiyama et al., 1992). When exposed 20–50 cGy of GCR containing iron ion, mouse trabecular and cortical bone showed 17% lower bone volume fraction and 4% lower thickness (Bandstra et al., 2009). These results demonstrate the negative effect of space IR on bone systems through mechanical blocking or damage, emphasizing the importance of conducting further study to safely explore the space beyond Earth.

## 3 Cancer Mechanomodulation

### 3.1 Tumor Microenvironment

**FIGURE 2:**
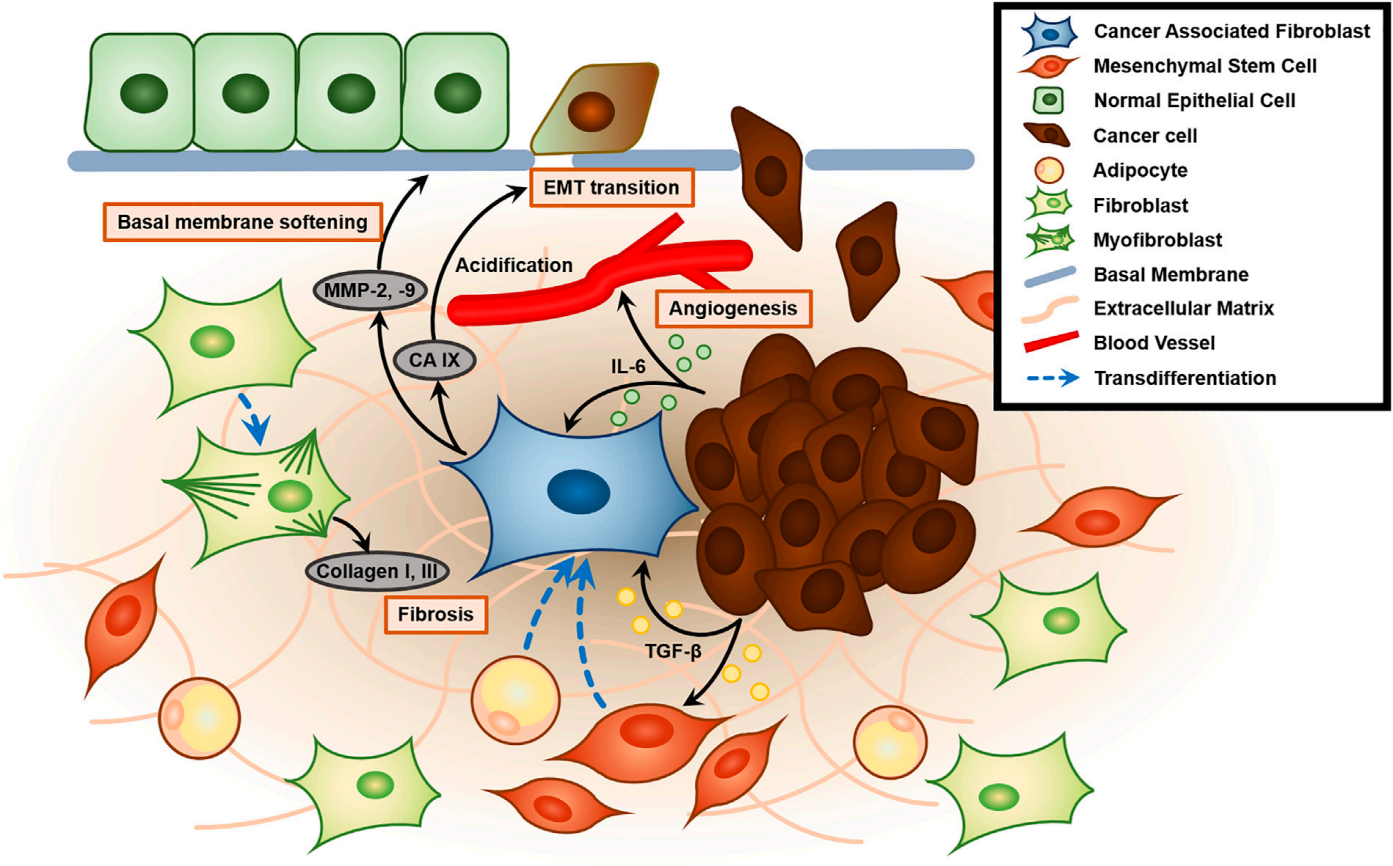
Role of the cancer-associated fibroblast (CAF) and tumor microenvironment (TME) in enhancing tumor malignancy. Tumor-secreted TGF-β activates and recruits mesenchymal stem cells and induces transdifferentiation into CAFs. Activated CAFs generate MMP-2 and MMP-9, which mechanically contract and remodel ECM to soften the basal membrane. Furthermore, CAF also secretes CA IX, which causes acidification of ECM and promotes epithelial-to-mesenchymal transition (EMT transition). Tumors also secrete IL-6, which plays a key role in angiogenesis. Contractile protein α-SMA in CAF promotes transdifferentiation of fibroblasts to myofibroblasts that excessively secrete collagen type 1 and 3, leading to fibrosis of the TME.

Cancer is a group of diseases characterized by dysregulation of cell division cycle caused by mutations or damage to tumor suppressor p53, which is central in regulating cell cycle and apoptosis, leading to abnormal proliferation (Vousden and Lane, 2007). Cancer cells actively interact with their microenvironment by chemical and physical signaling. Tumor cells and their neighboring microenvironments are highly interactive. Thus the changed physical settings in the space not only affect physiological features of tumor cells but their cellular and subcellular interactions with tumor microenvironment could be also altered. Since cancer cells reveal highly dynamic and bidirectional interactions with their microenvironment, we need to note the changes in following cell behaviors: 1) cell-cell contact and cell-ECM assembly, and 2) the biochemical mediators that modulate these contacts. These combinatorial effects of space can lower the immune responses in the tumor microenvironment. However, space-related signals have the possible effects of gene mutation, genomic instability, over-activated oncogenes, deactivation of tumor suppressors, as well as epigenetic modulation and abnormal metabolism due to deformed microenvironments. For instance, tumors affect their surroundings by secreting extracellular signaling molecules such as tumor growth factor-β (TGF-β), carbonic anhydrase IX (CA IX), and interleukin-6 (IL-6) that stimulate cancer progression (Figure 2) (Vidlickova et al., 2016). The tumor microenvironment (TME) includes the surrounding stromal cells, signaling molecules, blood/lymph vessels, and extracellular matrix (ECM). The tumor stroma itself is non-malignant, but it assists tumor growth, initiation, progression, chronic inflammation, angiogenesis, invasion, and metastasis, and configures approximately 90% of the TME (Denton et al., 2018). While the tumor stroma is composed of heterogeneous cells, the most abundant cell type is fibroblasts, particularly cancer-associated fibroblasts (CAFs), followed by endothelial cells, lymphocytes, pericytes, mesenchymal stem cells, and macrophages (Guo and Deng, 2018).

Morphologically similar to normal fibroblasts but greater than those cells, CAFs exhibit metabolic and transcriptomic activity, as termed “active fibroblasts” (Liu et al., 2019). CAFs are one of the main factors known to promote metastasis by interacting with cancer cells, but the criteria for distinguishing them from normal fibroblasts are still not well established due to their heterogeneous origin (Xing et al., 2010; Sahai et al., 2020). Up to 40% of CAFs exhibit the same markers as endothelial cells, such as PECAM/CD31, indicating its origin; the remainder show similarities to stromal cells, including bone marrow mesenchymal stem cells (MSCs) and adipocytes (Potenta et al., 2008; Lecomte et al., 2012; Bochet et al., 2013). Some molecules, e.g., alpha-smooth muscle actin (α-SMA), vimentin, fibroblast activation protein (FAP), metastasis-associated protein S100A4, and platelet-derived growth factor receptors PDGFRα/β, have been utilized as traditional biomarkers, although none are expressed exclusively by CAFs (Han et al., 2020). The expression of α-SMA depends on the concentration of TGF-β1, and is usually abundant in fibroblasts near a scar to assist in scar contraction (Huang et al., 2001; Shinde et al., 2017). The contractile protein α-SMA induces transdifferentiation of fibroblasts into myofibroblast, which excrete ECM compounds such as collagen I and III, as well as play a key role in wound closure and ECM contraction (Ruiz-Zapata et al., 2020). Myofibroblasts, also known as contractile cell, increase ECM rigidity by enhancing integrin binding, and cause tissue fibrosis in severe cases (Klingberg et al., 2013). The mechanism by which CAFs promote tumor progression is still not clear, but they are known to contribute through either paracrine signaling molecules or mechanical stimulation. For instance, carcinoma-produced TGF-β1 is known to play a key role in recruiting MSCs and transdifferentiating into CAFs. These MSCs produce hepatocyte growth factor (HGF) and tenascin C, which enhances tumor proliferation and promotes transformation into invasive phenotypes (Barcellos-de-Souza et al., 2016; Davies and Albeck, 2018).

In addition, IL-6 secreted from cancer cells facilitates angiogenesis and stromal changes to the CAF phenotype, increasing the levels of matrix metalloproteinase-2 and -9 (MMP-2 and MMP-9), which trigger ECM proteolysis (Nagasaki et al., 2014; Cancemi et al., 2020). These MMPs were expressed in neither epithelial cancer cells nor stroma-derived fibroblasts when each was solely cultured but detected in the fibroblasts co-cultured with the cancer. This result demonstrates that their expression in fibroblasts is induced by the intercellular interaction between epithelium and stroma, a key characteristic of epithelial-to-mesenchymal transitions (EMT) (Singer et al., 2002). The activated fibroblasts also produce CA IX, which lowers the pH of the TME, further increasing EMT, along with the invasiveness and metastatic ability (Fiaschi et al., 2013). Transfection of the tumor-associated isoform of CA IX into Madin-Darby Canine Kidney (MDCK) epithelial cells showed lower extracellular pH than did mock-transfected cells 48 h after incubation in hypoxia, in contrast to normoxia, which produced no significant difference in either group (Svastova et al., 2004). MDCK cells transfected with the mutant CA T443G, a known phosphorylation site activated by protein kinase A (PKA) in the intracellular domain of CA IX, exhibited restriction of the extracellular acidification during hypoxia. CA IX was concentrated and co-localized with sodium bicarbonate cotransporter-1 (NBC1), as well as the phosphorylated PKA substrates, in leading-edge membranes of the migrating hypoxic lung carcinoma A549 cells (Ditte et al., 2011). Because the transmembrane CA catalyzes the conversion of CO_2_ to bicarbonate and a proton on the extracellular side, these findings suggest that the coordination between the CA and NBC1 for regulation of bicarbonate metabolism in hypoxia is critical for extracellular acidification and cell migration. Conversely, TME acidification influences tumor physiology, as well. MCF7 mammary carcinoma cells cultured for 24 h at pH 6.0 showed more than 30% decrease in both cell viability and proliferation relative to those cultured at pH 7.2 (normal physiological condition). Both 24 and 48-h growth at the acidic pH exhibited more than 50% reduction in migration, examined as the percentage of the area of the cells covering the wound, compared to cells grown at normal pH (Ralph et al., 2020).

Moreover, 24-h incubation of MCF7 cells at pH 6.2 resulted in four-fold production of extracellular vesicles compared to that at pH 7.2, although no significant difference in size was observed (Ralph et al., 2020). Addition of the conditioned media containing these vesicles to non-transformed human fibroblasts at pH 6.2 showed a migration rate equivalent to that of cells cultured in nascent media at pH 7.2, five-fold higher than the acidic pH without the vesicle supplement (Ralph et al., 2020). These results imply the impact of extracellular pH on the exocytosis of tumorigenic cells during secretion of certain signaling molecules that activate the associated fibroblasts. Besides lowering pH, CAFs also use mechanical forces induced by actomyosin contractility to stretch the gaps of the basement membrane (BM), which was found to be independent of MMPs. Mesenteric basement membrane was co-cultured for 8 days with human colon cancer cells and CAFs (the latter derived from the patient with the low MMP expression), then laser-ablated to generate gaps within the membrane. 12 h after ablation, treatment with blebbistatin, an inhibitor of myosin II-dependent contractility, did not result in a significant change in the pore size, comparable to the untreated sole BM without the co-culture. In contrast, treatment of the co-cultured BM with the MMP inhibitor (GM6001) produced a notable increase in the pore size, similar to the extent of the untreated co-culture. Although BM stiffness was not affected by blebbistatin, the widening of gaps within the BM by actomyosin contractility is a contributor to cancer cell invasion (Glentis et al., 2017). Indeed, overcoming the stiffness of either the ECM or BM, which act as a barrier, is also essential for invasion.

ECM is composed mainly of collagens. Processing of collagens from procollagens by post-translational modification is required for their secretion (Winkler et al., 2020). It has been shown that the ECM of pancreatic ductal adenocarcinoma contains high levels of fibrillar collagens with incompletely cleaved C-terminal domains, suggesting a contribution of procollagens to tumor malignancy (Tian et al., 2021). The processing of pro-peptides is mediated by proteolytic enzymes such as lysyl oxidases (LOX), which are known to cross-link various ECM substrates and enhance the mechanical stability of ECM (Rosell-Garcia and Rodriguez-Pascual, 2018). Sensing stiffened ECM triggers the activation of focal adhesion kinase (FAK), a mechanosensing component of focal adhesion clustering (Xue and Jackson, 2015; Yeh et al., 2017) which stimulates its various downstream effectors through their phosphorylation. For example, activated FAK by vascular endothelial growth factor (VEGF) directly phosphorylates tyrosine residue of vascular endothelial cadherin (VEC-Y658), located at the cell junctions, which results in enhanced permeability of the endothelial cells by transmitting tumor cells through the attenuated cell-cell junctions (Jean et al., 2014).

To sum, tumors stiffen surrounding tissue by inducing fibrosis, and this stimulates tumor progression either by mechanically breaching BM or by secreting molecules that increase the permeability of cell barriers. The mechanism by which cancer cells penetrate stiff matrices better than normal cells is that cancer cells activate invadopodia when sensing matrix rigidity and contractile forces through Rho-associated kinase (ROCK) signaling (Jerrell and Parekh, 2016). Invadopodia are actin-rich protrusions of cancer that assist in the degradation and penetration of highly cross-linked BM by generating membrane type 1-MMP (MT1-MMP), a collagen-degrading protease, for invasion and metastasis (Enderling et al., 2008). Along with its proteolytic activity, MT1-MMP also exerts mechanical force to align the scaffolding protein Tsk5 and push the plasma membrane in the ECM direction (Ferrari et al., 2019). The coordination of physical regulation for invadopodia morphology, as well as the chemical modification of ECM, further promotes efficient invasion.

### 3.2 Cancer Invasion and Metastasis

Cancer invasion refers to the indication of malignancy, penetrating the neighboring tissue, and eventually causing metastases that form secondary tumors (Mareel and Leroy, 2003). Invasion is the primary step of metastasis, beginning with the loss of E-cadherin, which mediates cell-cell adhesion, resulting in dissociation from the primary tumor. E-cadherin expression is downregulated during carcinogenesis in Langerhans pancreatic islet β-cells (Perl et al., 1998). Furthermore, integrin β1 and MMP2 are known to be key factors in regulating cancer cell adhesion to ECM, and are therefore highly associated with invasion and metastasis (Wang et al., 2013). Invasive migration of tumor cells is classified into two types: collective migration and single cell migration (Krakhmal et al., 2015). Both migratory features are regulated by mechanical cues and cell-ECM adhesive integrin, but crowding, cohesion, and constraints are factors that determine collective migration (Chang et al., 2013). In collective migration, E-cadherin-mediated cell-cell junction domains within the group modify front-rear polarity. Although the junction is a prerequisite for establishment of collective migration, it is not necessary for its maintenance. Rather, the mechanical properties of each cell coordinate the front-rear polarity with the extension of a lamellipodium-based structure on the front cells (Jain et al., 2020). Polarization initiates the activation of cytoskeleton regulating Rho GTPase that results in transition of leader cells by utilizing shear stress on nearby cells and development of traction force to pull follower cells (Venhuizen and Zegers, 2017). Collective cell migration has been observed in the development and progression of breast, prostate, colorectal, and melanoma cancers, as well as in most squamous cell carcinomas (Krakhmal et al., 2015).

Single-cell migration also requires front-rear polarity within each cell, but differs from collective migration in that it does not require cell-cell junction (Jain et al., 2020). Single cell migration is further divided into two types of migratory modes: amoeboid and mesenchymal motions. Amoeboid movement is characterized by a tendency to appear in the soft matrix, weak cell-ECM adhesion, and high-velocity motion (Eichinger et al., 2005). In addition, cell-surface protrusion and blebs, which result from hydrostatic pressure generated in the cytoplasm by the contractile actomyosin cortex, appear during amoeboid locomotion by elevating myosin contractility through ROCK activation (Paluch and Raz, 2013). The suggested mechanism by which blebs play a critical role in migration is by determining the cytoplasmic flow direction to the protrusive site and anchoring the cell-ECM with E-cadherin at the neck of the bleb (Paluch and Raz, 2013). Single-cell cancer invasion effectively squeezes into the pores of the ECM in combination with lamellipodia and blebs, or in exclusive form. In contrast, mesenchymal migration is characterized by stress fibers and appear in a rigid matrix, where the cells displaying the mesenchymal migration deforms approximately 43% more ECM than cells following the amoeboid migration and performs strong myosin II-mediated anterior contraction through F-actin polymerization (Doyle et al., 2021).

In the metastasis cascade, cancer invasion is followed by angiogenesis that involves the formation of new blood vessels, controlled by oxygen and nutrient supply, into the hypoxic core of solid tumors, or invasion of other organs by forming circulating tumor cells (CTCs) (Zhao et al., 2017). The central signaling proteins of angiogenesis belong to the VEGF family, which consists of VEGF, VEGF-B, VEGF-C, VEGF-D, and placental growth factor (PlGF), and is strictly controlled by oxygen availability (Neufeld and Kessler, 2006). Each VEGF protein type differs in function, but shares the same mechanism of stimulating cellular responses, binding to three types of tyrosine kinase: VEGF receptors VEGFR1 (VEGF receptor 1), VEGFR2, and VEGFR3, which are embedded in the cell membrane and transduce intracellular signaling pathways through transphosphorylation (Pandey et al., 2018). VEGF can bind to VEGFR1 and 2, activating angiogenesis, lymphangiogenesis, vascular permeability, and vascular homeostasis through phosphotidylinositol-3 kinase/protein kinase B (PI3K/Akt) when bound to VEGFR2 (Pandey et al., 2018). Besides VEGF, mechanical stress can also mediate tumor angiogenesis. For example, an increase in ECM density and stiffness promotes an angiogenic sprouting response from multicellular spheroids upon MMP activation (Bordeleau et al., 2017). This was due to the expression of protein kinase C beta type 2 (PCK βII), a pro-angiogenic factor that contributes to cell proliferation and motility through mitogen-activated protein kinase (MAPK) activation, which increases more than 2-fold in stiffer matrices (Bordeleau et al., 2015). Furthermore, stiffness downregulates the VEGF 165b isoform, which has anti-angiogenic function through competitively inhibiting angiogenic VEGF binding to VEGFR 2; it also has a distinguishing C-terminal exon coded for SLTRKD (Bordeleau et al., 2015).

### 3.3 Cancer Mechanomodulation

Extracellular physical cues induced by ECM deformation and alteration of ECM rigidity are transmitted through integrins, cytoskeletons, and the nuclear membrane (Broders-Bondon et al., 2018). Mechanical tension promotes attachment to specific molecules in the extracellular matrix (ECM), and Yes-associated proteins (YAP) are essential for the adhesion-mediated pathways, activated by phosphoinositide 3-kinases (PI3Ks) (Spencer et al., 2021). Specifically, adhesion to extracellular fibronectin induces YAP translocation in the nucleus through the FAK-Src-PI3K-PDK1 pathway (Kim and Gumbiner, 2015). Cells plated on adhesive islands of ECM tend to proliferate, whereas those on the narrow island go through apoptosis (Dupont et al., 2011). However, cells lacking nuclear translocation of YAP consistently showed the reduced proliferation, as well as the enhanced apoptosis rate, regardless of the island size (Dupont et al., 2011). Regulation of proliferation and apoptosis is dependent on the cell adhesion-mediated mechanotransduction that results in the nuclear YAP translocation. When the tumor reaches the late progression stage with continuous proliferation, hypervascularization and compactness increases, leading to increased interstitial fluid pressure, which can be elevated up to 10 times compared to the normal state (Heldin et al., 2004). Growth-induced solid stress compresses in the direction of the blood vessel, blocking blood flow and restricting nutrition and oxygen accessibility. Such mechanical forces induce hypoxia, which is a prominent factor in malignant cancers (Li et al., 2021).

The family of hypoxia-inducible factors (HIFs) is composed of α and β subunits: HIF-1α contains a proline residue (P564) that is hydroxylated by the prolyl hydroxylase (HIF-PH), which utilizes oxygen as a co-substrate. The von Hippel-Lindau tumor suppressor (pVHL) ubiquitinates the hydroxylated subunit, leading to its degradation (Jaakkola et al., 2001). However, under hypoxic conditions, HIF-1α is stable, so the entire heterodimer serves as a transcription factor for target genes that control the metabolic switch from oxidative phosphorylation to anaerobic fermentation (Seagroves et al., 2001). As a result, HIF1 enables tumors to adapt to hypoxia triggered by the excessive respiration of proliferative cells. Hypoxia-activated HIF-1α was also shown to induce the expression of collagen prolyl 4-hydroxylases (P4HA1 and P4HA2) and procollagen-lysine, 2-oxoglutarate 5-dioxygenases (PLOD2) in breast cancer-associated fibroblasts that promoted the ECM stiffness, where hydroxyproline-collagen expression was diminished in the fibroblast culture upon shRNA-knockdown of P4HA1 and P4HA2 (Seagroves et al., 2001). Although PLOD2 did not affect the amount of the extracellular hydroxylated collagen as much as those collagen prolyl hydroxylases, it promoted construction of the integrated collagen fiber under hypoxia (Gilkes et al., 2013a). Co-localization of PLOD2 with fibrillar collagens supports its function as the inter-fiber crosslinking, which leads to increase in the matrix stiffness as well as invasiveness of the tumors (Gilkes et al., 2013b). The HIF1-induced collagen secretion and organization under hypoxic condition entail the stiffness gradient in ECM, which could play a role in migration of cancer cells.

Cell placed on compliant matrix migrate to the rigid matrix, a tendency termed durotaxis that depends on the focal adhesion turnover and detachment of the rear edge from the matrix by myosin 2–mediated contractility (Maiuri et al., 2015; Lachowski et al., 2017). In particular, myosin-IIB (MIIB) showed increased concentration and polarization at the rear side of the mesenchymal stem cell on the stiff matrix (Raab et al., 2012). Upon activation of the myosin light chain (MLC), MIIBs self-assemble with their C-terminal domains, leading to the formation of actomyosin filaments that inhibit frontal protrusion. In contrast, another isoform, myosin-IIA (MIIA), consists of filaments in a distinct region of the MIIB to support the anterior protrusion. More specifically, MIIB was found to be incapable of independent formation in the absence of MIIA expression, unlike the opposite case, in which MIIA formed filaments thinner than those from both expression, indicating initial conjugation of actin filaments with MIIA followed by the subsequent assembly of MIIB (Vicente-Manzanares et al., 2008). To differentially regulate MIIA and MIIB for the cytoskeletal assembly, the function of their upstream activators should be reactive to ECM stiffness: the matrix rigidity as measured by the fibronectin density was proportional to the degree of cellular MLC phosphorylation, along with the traction force within the cytoskeleton (Polte et al., 2004). Each myosin activator has distinct substrates, and the pair co-localizes on a specific site of the cytoskeleton: Rho-associated kinase (ROCK) co-regulates the physical properties, including the viscoelasticity, of the central actomyosin fibers with MIIA, whereas myosin light chain kinase (MLCK) co-regulates peripheral fibers with MIIB (Chang and Kumar, 2015). Moreover, ROCK was shown to generate sufficient force within the actomyosin fibers to remodel the ECM in the absence of metalloprotease activity (Wyckoff et al., 2006). To sum up, the front-rear polarization of cells depends on both the spatial and functional differentiation of the action of ROCK with MIIA and MLCK with MIIB.

## 4 Cancer Progression in the Space Environment

### 4.1 Decompression and Cancer

**FIGURE 3:**
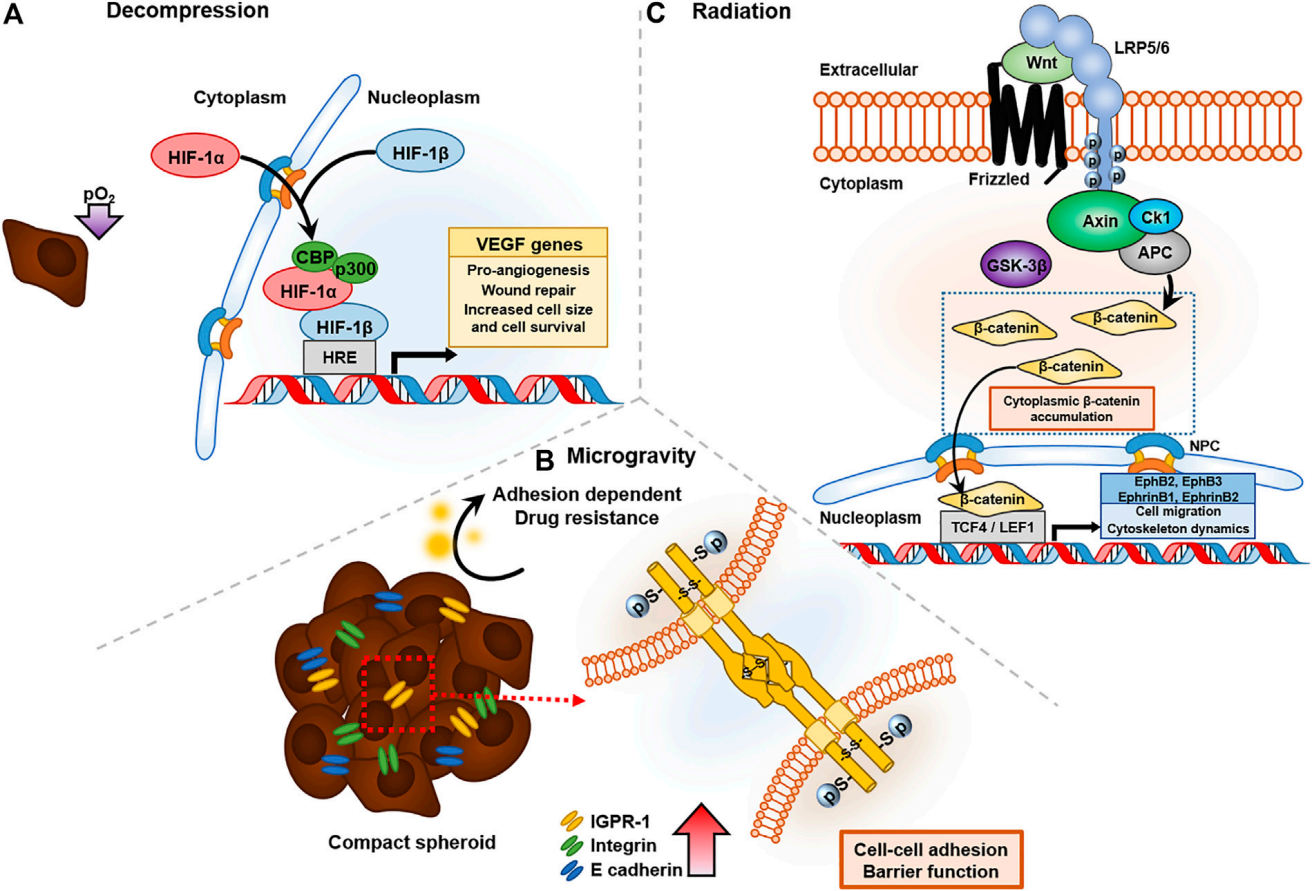
Cancer affected by three representative altered environmental conditions in space: decompression, radiation, and microgravity. **(A)** Decompression of oxygen activates HIF-1α expression and forms a DNA binding complex with CBP/p300 to VEGF gene transcripts, which have pro-angiogenesis and wound-repair functions. **(B)** Microgravity upregulated the expression of cell-to-cell junction proteins, such as IGPR-1 and E-cadhrin, which have barrier functions in drug resistance. **(C)** Radiation in space triggers the Wnt signaling pathway, and accumulated β-catenin bind to T-cell factor/lymphoid enhancer factor family (TCF/LEF), leading to transcription of EphB/EphrinB, which regulates cell migration and cytoskeletal dynamics.

Decompression in space causes hypoxia either by induced nitrogen evaporation or hypobaric conditions. When sufficient oxygen is not supplied, cells express hypoxia inducible factor-1α (HIF-1α), a key oxygen-regulated transcriptional activator that assists in adaptation of tumor cells to scarce oxygen by upregulating the transcription of genes related to tumor cell survival, proliferation, angiogenesis, and anti-apoptosis. Under exposure to low-pressure atmospheric conditions, such as pO_2_ at an altitude of 5,000 m, M2-like tumor-associated macrophages (TAMs) activate and significantly increase the population of cervical cancer cells in response to upregulated expression of neuropilin-1 (Nrp-1) and CA IX (Chen et al., 2019). TAM is known to create an immunosuppressive TME and facilitate metastasis and angiogenesis (Lin et al., 2019). In addition, hypobaric hypoxia increases radio resistance in space by repairing damaged DNA radicals with an antioxidant that contains sulfhydryl groups (Wang et al., 2019). When cervical cancer cells are exposed to high IR, tumor size and cell survival are higher under hypoxia than under normoxia (Fu et al., 2015). This is due to protection by HIF-1α inhibition of the irradiation-induced apoptotic protein, p53, a mechanism that has been verified by silencing HIF-1α (Fu et al., 2015). Structural recruitment of the C-terminal transactivation domain (CTAD) of HIF-1α and cysteine/histidine rich domain 1 (CH1) of CBP/p300 leads to transcription of VEGF genes, prominent for pro-angiogenic functions in tumor progression (Figure 3A) (Kwon et al., 2012). VEGFs known to stimulate tumor progression such as angiogenesis and metastasis significantly decrease the survival rate of gastric cancer patients by increasing cancer cell survival rate (Ozdemir et al., 2006).

Under hypoxic conditions, energy metabolism through lactic acid fermentation domains, rather than the tricarboxylic acid (TCA) cycle in mitochondria, results in acidosis. Sensing acidification of ECM, carcinoma cells increase expression of the lactate/H+ symporter monocarboxylate transporter (MCT) family, consisting of MCT1, MCT2, MCT3, and MCT4 (Kim et al., 2015), where, MCT1 and MCT4 contribute to cancer malignancy by shuttling lactate within tumor, which serves as a respiratory fuel in tumor metabolism (Gao et al., 2015). TME acidosis increases myeloid-derived suppressor cells (MDSCs), heterogenic clusters of immune cells during cancer, by stimulating neutrophils through activating PI3K/Akt, which regulates proliferation, and ERK/MAPK pathways, which produce proinflammatory cytokines (Martinez et al., 2006; Gabrilovich and Nagaraj, 2009). Among four classes of PI3Ks (class I, class II, class III, and class IV), class I PI3K exhibits oncogenic activity by converting phosphatidylinositol 4,5-bisphosphate (PI4,5P2) to phosphatidylinositol 3,4,5-trisphosphate (PIP3), and recruited PIP3 activates Akt that induces oncogenic activity by inducing cell growth, proliferation, and resisting to apoptosis (Zhao and Vogt, 2008). Taken together, these results indicate that hypoxic condition during deep-space exploration increase the survival rate of cancer cells.

### 4.2 Microgravity and Cancer

Microgravity suppresses immune activity and increases the risk of cancer (Jhala et al., 2014). Even independently of immune cells, microgravity increases cancer survival and progression. Microgravity is sensed by cells and stimulates biochemical changes, which are classified as mechanical stressors that can alter cancer progression (Aventaggiato et al., 2020). For instance, when MCF7 cells are exposed to reduced gravity, they exhibit rearrangement of actin and microtubule architecture (Nassef et al., 2019), and they form multicellular spheroids after 24 h (Kopp et al., 2016). Using a random positioning machine, which is a two-axis form of the clinostat that reduces the gravity vector averaging to zero (Wuest et al., 2015), could downregulate gene expression patterns, such as those of vascular endothelial growth factor-A (VEGFA), vascular endothelial growth factor receptor 2 (FLK1), caspase-9 (Casp9), caspase-3 (Casp3), and protein kinase C alpha (PRKCA), which interfere with 3D cell aggregation (Kopp et al., 2018). However, VEGF release was clearly upregulated in MCF-7 cells under microgravity conditions. These conflicting results indicate that some factors may act differently under stimulated and real microgravity, underscoring the need for future research on real microgravity. A similar phenomenon was observed in thyroid cancer cells (FTC-133), and researchers found that decreased integral membrane protein caveolin-1, due to stimulated microgravity, enhances spheroid forming (Riwaldt et al., 2015). Cancer cells form spheroids to resist cytotoxic and anti-cancer drug effects, which is termed “multicellular resistance” when tested on adenocarcinoma cell lines. The mechanisms of reversible MCR are based on contact resistance, because forming 3D aggregates minimizes tumor cell-cell contact and cell-ECM contact, and cells with high expression of junction proteins such as E-cadherin and integrin exhibit drug resistance (Desoize and Jardillier, 2000). In human colorectal cancer cells, immunoglobulin-containing and proline-rich receptor-1 (IGPR-1), which mediates endothelial barrier function and cell-to-cell interaction, is upregulated and increases survival under chemotherapeutics such as SB203580, a p38 inhibitor, by causing multicellular aggregation (Figure 3B) (Woolf et al., 2017).

Increased CD44^+^ and CD133^+^ expression was observed under both simulated microgravity and 3D culture in non-adherent 96-well plates (Arun et al., 2019). CD44 is a multifunctional transmembrane protein that mainly mediates tumor metastasis, as well as cell-cell interaction; various metastatic cancer cells exhibit increased expression of CD44 and its isoform (Basakran, 2015). CD133, also known as prominin-1, is known to be positively correlated with chemoresistance, metastasis, invasion, and stemness of tumors (Zhang et al., 2013). Colorectal stem cells highly expressing CD44^+^/CD133^+^ exhibited carcinogenic proliferation and cell cycle characteristics compared to those with low expression (Zhou et al., 2016). However, culturing under simulated microgravity exhibited nuclear localization of YAP and abnormally increased the size of cancer cells, while 3D culture under normal gravity did not produce a significant difference. YAP expression induces four Yamanaka factors: a significant increase in octamer binding transcription factor 4A (OCT4A), and slight upregulation of sex determining region box 2 (SOX2), Nanog (homeobox protein), and NKx-2.5 (NK2 homeobox 5). These upregulated factors are known to play a significant role in maintaining the stemness of cancer cells by disturbing the tumor-suppressive Hippo pathway (Basu-Roy et al., 2015). These data strongly support the opinion that space microgravity increases the malignancy of cancer cells, maintains stemness, and leads to an abnormal tumor size. The genetic expression of thyroid cancer cells (UCLA RO82-W-1 cell line), grown as an adherent phenotype under normal gravity and forming spheroids under microgravity, was compared. The result was the upregulation of MMP3 and connective tissue growth factor (CTGF), and downregulation of plasminogen activator inhibitor-1 (PAI-1). CTGF-rich TME has been implicated in increased malignancy in gliomas (Edwards et al., 2011). Space microgravity is known to increase CTGF gene expression, which detaches surface adhesion and induces 3D aggregation (Pietsch et al., 2013). Furthermore, downregulation of PAI-1 contributes to spheroid formation because plasminogen accumulation is disturbed (Riwaldt et al., 2016). Further data from the FTC-133 cell line revealed that microgravity generated using a random-positioning machine decreased the expression of CAV1 and connective tissue growth factor (CTGF) after 74 h of simulation in microgravity-induced spheroids when compared to adherent cells under both normal and microgravity environments (Warnke et al., 2014). CTGF expression can be induced by mechanical stimulation, such as hypertension, and plays an essential role in tissue remodeling and fibrosis by activating myofibroblast activation (Lipson et al., 2012).

Adipocytes are closely adjacent to breast cancer cells, constituting breast tissue and cancer metastases to other organs after lipid accumulation (Le et al., 2009). Therefore, it is evident that obese patients whose adipocytes are both hypertrophic and hyperplastic tend to have more aggressive cancer progression (Tan et al., 2011). Space microgravity significantly increased the gene expression of CCAAT Enhancer Binding Protein Beta (CEBPB), a key regulator of adipogenic differentiation, by directly inducing expression of peroxisome proliferator activated receptor gamma (PPARγ2) (Barlier-Mur et al., 2003; Zhang et al., 2018). Furthermore, microgravity switches the morphology of adipose-derived stem cell (ADSCs) from a flat spindle to a round phenotype, resulting from the destruction of F-actin and tubulin structures, similar to MCF-7 cells (Ebnerasuly et al., 2017). Increased connective tissue growth factor in ADSCs under simulated microgravity enhanced the expression of collagen type I and III, which contribute to cancer angiogenesis mainly through TME fibrosis (Ebnerasuly et al., 2017).

Space gravity also upregulates matrix metalloproteinase (MMP) 1 by approximately 12.94-fold in bone, promoting bone erosion through upregulation of cysteine-rich angiogenic inducer 61 (CYR61) via the discoidin domain receptor 2-matrix metalloproteinase-1 (DDR2-MMP1) signaling pathway (Blaber et al., 2013; Huang TL. et al., 2019). CYR61, also known as cellular communication network family member 1 (CCN1), interacts with cell-surface integrin receptors and regulates cell adhesion, migration, proliferation, and differentiation induced by transphosphorylation of DDR2 and activator protein 1 (AP-1) (Huang TL. et al., 2019). In summary, space microgravity activates factors that promote fibrosis and adipogenic differentiation, which contributes to cancer proliferation and metastasis.

### 4.3 Ionizing Radiation and Cancer

Radiation is widely used for cancer treatment because it transfers energy intense enough to destroy cancer cells. However, similar to other cancer treatments, radiation therapy also causes side effects, such as fibrosis, increased *in vivo* toxicity, and apoptosis (Ratajczak et al., 2013). Irradiation also induces mechanical changes. For example, the surface of the enamel and the underlying dentin increased in microhardness by 1.2-fold when irradiated with a total dose of 60 Gy (de Siqueira Mellara et al., 2014). However, the indentation hardness of the deep enamel, dentinoenamel junction, and deep dentin, as well as the pulp chamber, was not affected by irradiation, indicating the limit of penetration. Likewise, irradiation induces stiffening in breast cancer, with a significant increase in collagen production, and may lead to organ failure. This was shown through co-culturing breast cancer MCF-7 cells in Matrigel with MRC-5 fibroblasts for *in vivo* mimicking, and it was found that upregulated TGF-β induces fibrogenesis through activation of myofibroblasts by canonical Wnt signaling pathways under 2 Gy of radiation exposure (Yakavets et al., 2020)**.** Even low-dose, heavy-ion radiation triggers persistent stress signaling, such as binding β-catenin, downstream of the Wnt signaling pathway, to the T-cell factor/lymphoid enhancer factor family (TCF/LEF) binding site of EphB/EphrinB promoters, inducing DNA damage and chronic oxidative disease, and finally leading to senescence-associated secretory phenotype (SASP) (Figure 3C) (Kumar et al., 2018).

Space radiation, which contains highly ionizing heavy ions such as iron, silicon, and calcium, downregulates Cdc42, myosin light-chain kinase (Mlck), Par3, and E-cadherin, and increase Rock1; these are factors that contribute to cytoskeletal remodeling, migration, and cell-polarity dynamics (Kumar et al., 2018). Downregulation of E-cadherin is the primary step in carcinogenic metastasis and weakens cell-cell adhesion, resulting in dissociation from the primary tumor. Ionizing radiation (IR) is known to stimulate radiation-induced inflammatory responses by activating transcription factors, e.g., NF-κB, STAT-3, and HIF-1, which modulate the TME and promote cancer development (Mckelvey et al., 2018). SASP generates IL-6, the proinflammatory cytokine, from senescent fibroblasts, resulting in enhanced invasiveness and facilitated progression of breast cancer cells (Coppe et al., 2010). These results prove that radiation induces fibrosis in cancer and surrounding tissue, either by secreting cytokines associated with cytoskeletal remodeling, including collagen production and junction increase, or by inducing proinflammatory reactions.

Histopathologically, an increased number of profibrotic foci occurred, with 17 upregulated and nine downregulated ECM-related genes, in connective tissue in mice during spaceflight (Tian et al., 2010). Profibrotic foci indicate increased ECM stiffness, which is associated with regulation of cancer progression (Burgess et al., 2016; Bregenzer et al., 2019). For instance, the risk of lung carcinoma increases *via* idiopathic pulmonary fibrosis (IPF), which is characterized by fibrotic foci (Park et al., 2001; Burgess et al., 2016). Increased ECM stiffness mediates phenotypic transition of cancer stem into more metastatic forms in colorectal cancer (Tan et al., 2019) and breast cancer (Seewaldt, 2014) by reducing phosphatase and tensin homolog (PTEN) expression, thereby upregulating the phosphoinositide 3-kinase (PIP3)-AKT pathway (Mouw et al., 2014). Furthermore, ECM stiffening elevates levels of activated β1 integrin, pY397 FAK, and pS19 MLC, which tend to increase with mechanosignaling and promote human breast cancer progression (Acerbi et al., 2015). However, the mechanism by which radiation induces profibrotic foci and elevates ECM stiffness has not been fully elucidated. Further research to reveal mechanisms should be conducted for future space exploration, since the IR effect is directly related to both chronic and acute diseases.

### 4.4 Space Environment Effect on Aging and Cancer Progression

While the most well-known physiological phenomenon in long-term spaceflight is decreased skeletal muscle mass, 14.5% of telomere prolongation compared to preflight has also been reported by NASA (Garrett-Bakelman et al., 2019). The length of telomeres shortens with every mitotic cell division by 50–200 bp, which is known as telomere attrition; mice with short telomeres have shorter lifespan, organismal aging, and a higher risk of age-related pathologies (Bernardes de Jesus et al., 2012). In contrast, improved metabolic parameters, less senescence DNA damage, and increased longevity were observed in mice with hyperlong telomeres (Bernardes de Jesus et al., 2012; Munoz-Lorente et al., 2019); thus, short telomeres are considered a hallmark of aging (Lopez-Otin et al., 2013). Telomere elongation was discovered earlier in short-term spaceflight in *Caenorhabditis elegans*, in which the length increased from ∼7 to ∼9 kb (approximately 29%) when measured by single telomere length analysis (STELA) (Zhao et al., 2006). The space environment also downregulates expression of genes related with neuronal or endocrine signaling (gar-3, unc-17, cha-1, F57A8.4, glc-4, shk-1, and ins-35) in *C. elegans*, and inactivation of these genes increased lifespan on the ground (Honda et al., 2012).

Aging and age-related diseases, such as cancer, seem to be suppressed during spaceflight; however, telomere length rapidly (within 48 h on Earth) became shorter than preflight, with severe DNA damage in astronauts after long-term space missions (Garrett-Bakelman et al., 2019; Luxton et al., 2020b). Plasma concentrations of interleukins (IL-1a, IL-2, IL-4, IL-5, IL10), chemokines (CCL4, CCL5, CXCL5), and vascular endothelial growth factor-1 (VEGF-1) strongly exhibited the same increasing and decreasing aspects as mean telomere length during the entire spaceflight mission, i.e., preflight, during the flight, and post flight (Luxton et al., 2020a). Approximately 80% of human cancer cells activate telomerase to elongate telomeres and promote unlimited replication. In contrast, telomere shortening suppresses the formation and replication of cancer, but promotes tumor malignancy (Hirashima et al., 2013). For example, in the dysfunctional telomere group, *mTerc* ^*−/−*^ mice activate the p53-dependent senescence pathway, discarding the p53-dependent apoptosis pathway, thereby suppressing tumorigenesis (Deng et al., 2008). Telomere length is known to be associated with cancer incident and mortality. Mice with short telomeres exhibited incident and mortality rates of 22.5 and 10.6%, respectively, while the group with longer telomeres displayed rates of only 5.1 and 0.7%, respectively (Willeit et al., 2010). This phenomenon is supported by the suggestion that shortening of telomeres reduces protective function and induces dysfunction, which eventually causes genome instability by increasing chromosome fusion, anaphase bridge, and nonreciprocal translocation frequency (Hackett et al., 2001). The space environment has a high potential to increase the cancer incidence rate by microgravity-induced ECM stiffening, hypobaric hypoxia, increased adipocytes, and irradiation. The fact that telomeres elongate in space could be due to telomerase activation by space-induced cancer cells.

The impact of the space environment on biological characteristics regulating oncogenesis and tumor growth such as redox stress, telomeres, mutagenesis, and epigenetic regulations is less studied. Fundamental features of space biology includes oxidative stress, DNA damage, mitochondrial dysregulation, genetic and epigenetic modification, telomere alterations, and microbiome shifts as well as their associated health risks of space exploration (Afshinnekoo et al., 2020). Accordingly, aerospace medicine highlighted in the articles. The health hazards associated with spaceflight are collectively influenced by space radiation, microgravity, containment/isolation, distance from Earth, and a hostile/closed environment. Since the health conditions affect physiological systems in the human body, including the central nervous, cardiovascular, musculoskeletal, immune, and hepatic systems, the space flight could result in the disruption of circadian rhythms, increased risk of cancer, and genetic mutations. For instance, recently, space technology merging the 3D organoid technology has been applied to study early mutational events in human DNA due to spaceflight exposure (Larose, 2020). Thus the development of effective counter-measures and health systems play a key role to explore the next phase of space exploration. Aerospace medicine will therefore advantage from longitudinal profiling, multi-omic, that captures the collective effects from exposures throughout multiple risks and interactions among multiple organ structures and biological functions.

## 5 Conclusion Remark

Unlike on Earth, various environmental factors are altered during spaceflight. In particular, reduced gravitational force, decompressed air molecules, and high energy heavy-ion irradiation are the most prominent current physiological concerns during spaceflight, as they are known to increase the risk of illnesses such as cancer and cardiovascular disease. Accumulating evidences demonstrate that tumors are highly affected by external physical factors, and such mechanical cues promote cancer progression during carcinogenesis, proliferation, invasion, and metastasis, but there is no detailed information on how cancer cells in the human body are affected by altered external environments in space. Advances in experimental methods and analysis tools allowed many researchers to further study altered environmental cues during spaceflight. Microgravity, decompression, and ionizing radiation in the space environment regulate cancer malignancy by altering the tumor microenvironment, such as fibrosis, proinflammatory response, and angiogenesis. Decompressed pO_2_—translocate HIF-1α into the nucleoplasm to inhibit the apoptotic protein p53 and to transcribe VEGF genes, which are the main factors to promoting angiogenesis, wound repair, cell survival, and proliferation. Furthermore, high-energy radiation such as GCR and SPE upregulates TGF-β, which induces fibrosis of the TME through activation of myofibroblasts. Rigid ECM stimulates MSCs in the TME to secrete hepatocyte growth factor (HGF) and tenascin C, factors that promote tumor proliferation and transform into invasive phenotypes. Tumor cell survival is increased during spaceflight by telomere elongation, as well as apoptotic pathway inhibition and compact spheroid formation. For example, MCF7 breast cancer cells under microgravity remodeled cytoskeletal organization and formed multicellular spheroids by increasing cell-cell junctions. The formation of spheroids minimizes cancer cell exposure to the cytoplasm and enhances barrier function, providing adhesion-dependent cytotoxic resistance.

Similar to other chronic diseases such as bone/muscle atrophy and cardiovascular disease, cancer incidence is also a major side effect of long-term space exploration. Since significant alteration of mechanical cues in the space environment may affect the human body, further investigation of space mechanobiology and its effect on both acute and chronic diseases should be conducted. Comprehension of mechanotransduction processes compared to Earth is one of the most important challenges in space mechanobiology. Thus, more complex and delicate 3D *in vivo* mimicry of exposure to space conditions—not only microgravity, irradiation, and decompression, but also other possible mechanical cues that differ from those on Earth—are the key to human advancement into space.
